# Leveraging genome-enabled growth models to study shoot growth responses to water deficit in rice

**DOI:** 10.1093/jxb/eraa280

**Published:** 2020-06-12

**Authors:** Malachy T Campbell, Alexandre Grondin, Harkamal Walia, Gota Morota

**Affiliations:** 1 Department of Animal and Poultry Sciences Virginia Polytechnic Institute and State University Blacksburg, VA, USA; 2 Department of Agronomy and Horticulture University of Nebraska-Lincoln, Lincoln, NE, USA; 3 UMR DIADE, Université de Montpellier Institut de Recherche pour le Développement (IRD) Montpellier, France; 4 CSIRO Agriculture and Food, Australia

**Keywords:** Aquaporin, drought, genome-wide association study, genomics, growth model, phenomics, rice

## Abstract

Elucidating genotype-by-environment interactions and partitioning its contribution to phenotypic variation remains a challenge for plant scientists. We propose a framework that utilizes genome-wide markers to model genotype-specific shoot growth trajectories as a function of time and soil water availability. A rice diversity panel was phenotyped daily for 21 d using an automated, high-throughput image-based, phenotyping platform that enabled estimation of daily shoot biomass and soil water content. Using these data, we modeled shoot growth as a function of time and soil water content, and were able to determine the time point where an inflection in the growth trajectory occurred. We found that larger, more vigorous plants exhibited an earlier repression in growth compared with smaller, slow-growing plants, indicating a trade-off between early vigor and tolerance to prolonged water deficits. Genomic inference for model parameters and time of inflection (TOI) identified several candidate genes. This study is the first to utilize a genome-enabled growth model to study drought responses in rice, and presents a new approach to jointly model dynamic morpho-physiological responses and environmental covariates.

## Teaser

A genome-enabled growth model reveals a trade-off between early vigor and drought tolerance, and identifies an aquaporin as a potential candidate for regulating temporal drought responses.

## Introduction

Rice is one of the most important food crops and is a major source of food security for >3.5 billion people worldwide. Adequate water availability is essential for proper vegetative growth and grain development. Approximately 40 Mha of rainfed rice is grown worldwide, with the majority of production being concentrated in developing nations (Singh and Singh, 2000). Erratic precipitation events, as well as the increased competition for fresh water for non-agricultural uses, has become a major constraint for rice production (Korres *et al.*, 2017).

Given the socioeconomic impacts of water limitations, improving drought tolerance is a major target for breeding programs. However, the multiple and often unpredictable drought stress scenarios in drought-prone environments makes improvement of drought tolerance in rice challenging. Further, traits that are important for adaptation to limited water availability, particularly morpho-physiological traits, are complex and often have low heritability (Kamoshita *et al.*, 2008). These characteristics impede the discovery of loci that confer large effects on the phenotype, and limit the utility of marker-assisted selection for improving drought tolerance.

Recent advances in phenomics and genomics have offered new tools for discovering and quantifying traits associated with drought adaptation and their genetic basis (Berger *et al.*, 2010; Furbank and Tester, 2011; Araus and Cairns, 2014). Access to high-throughput, image-based phenomic systems in the public sector has allowed researchers to non-destructively measure traits of interest for large populations in highly controlled greenhouse or field environments. These platforms provide an effective means to study temporal developmental and/or physiological processes and assess how these processes are influenced by environmental factors such as drought (Berger *et al.*, 2010). Several studies have leveraged functional approaches to describe these temporal phenotypes using simple mathematical models, and leveraged genetic mapping approaches to identify loci that may affect trait trajectories (Ma *et al.*, 2002, Malosetti *et al.*, 2006; Das *et al.*, 2011; Bac-Molenaar *et al*., 2015, 2016; Campbell *et al.*, 2015).

In addition to the temporal phenotypes generated with these platforms, many field- and greenhouse-based platforms also collect high-resolution environmental data (Tardieu *et al.*, 2017). These data provide additional insight into how temporal physiological and/or morphological responses are influenced by environmental conditions. Several studies have used these traits as covariates in the conventional genomic prediction frameworks to increase prediction accuracies for agronomic traits such as yield (Aguate *et al.*, 2017; Montesinos-López *et al.*, 2017; Sun *et al.*, 2017; Krause *et al.*, 2019). However, with these approaches, secondary phenotypes are utilized as linear predictors without directly considering how they give rise to the observed phenotype.

Process-based eco-physiological models seek to predict outcomes by explicitly modeling the interaction of biological processes with environmental covariates (Batchelor *et al.*, 2002; Ittersum *et al.*, 2003; Parent and Tardieu 2014). However, a major disadvantage of these models is that genotypic variation is often unaccounted for or not optimally utilized in the predictions (Onogi *et al.*, 2016). Thus, their application in genomic prediction or inference studies is limited. Several studies have sought to integrate crop growth models with established quantitative genetic frameworks (Technow *et al.*, 2015; Onogi *et al.*, 2016; Wang *et al.*, 2019). For instance, Technow *et al.* (2015) used an approximate Bayesian computation framework to integrate crop growth modeling and whole-genome prediction to predict yield in maize. They showed a clear advantage of the genome-enabled crop growth model over the conventional genomic prediction approach using simulated data. More recently, Onogi *et al.* (2016) leveraged a crop growth model to predict heading date in rice that integrated the phenological model proposed by Yin *et al.* (1997) and implemented by Nakagawa *et al.* (2005) with a whole-genome prediction using a hierarchical Bayesian approach. The hierarchical Bayesian approach outperformed conventional genomic prediction models as well as approaches that fit the crop growth model and genomic prediction model in separate steps. The advantage of the integrated approaches proposed by Technow *et al.* (2015) and Onogi *et al.* (2016) is that model parameter estimates are informed by the genomic relationships among the accessions, which can improve the accuracy of the parameter estimates. Moreover, since these approaches are based on a Bayesian whole-genome regression framework, marker effects are predicted, facilitating marker-level associations with model parameters. However, to date no studies have leveraged these genome-enabled crop growth models for biological inference, or to elucidate the genetic loci that influence model parameters.

In the current study, we sought to leverage the frameworks developed by Onogi *et al.* (2016) to study the effects of water deficit on shoot growth trajectories for a diverse set of rice accessions. To this end, accessions were subjected to drought stress [20% field capacity (FC)], and shoot growth was quantified over 21 d using an image-based phenomics platform. A corresponding set of accessions was maintained under optimal water conditions (90% FC). The automated phenotyping system allowed daily water use (WU) for each accession and soil water content to be estimated. Together, these data were used to develop a novel growth model that models shoot growth trajectories as a function of soil water content and time. This growth model was integrated into the hierarchical Bayesian framework of Onogi *et al.* (2016) to elucidate the genes underlying model parameters. This approach provides a biologically meaningful framework that simultaneously (i) models the inter-relationship between growth rate and soil water availability; (ii) estimates quantitative trait loci (QTLs) effects for model parameters; and (iii) provides genetic values for model parameters that can be used for genetic evaluation.

## Materials and methods

### Plant materials and greenhouse conditions

A subset of the Rice Diversity Panel 1 was used in this study (Zhao *et al.*, 2011). Seed preparation was performed following Campbell *et al.* (2015). Briefly, seeds were surface sterilized with Thiram fungicide and were germinated on moist paper towels in plastic boxes for 3 d. Three uniform seedlings were selected and transplanted to pots (150 mm diameter×200 mm height) filled with ~2.5 kg of UC Mix. Square containers were placed below each pot to allow water to collect. Temperatures in the greenhouses were maintained at 28/26.0 °C (day/night), and relative humidity was maintained at ~60% throughout the day and night.

### Experimental design

A total of 378 accessions were phenotyped at the Plant Accelerator, Australian Plant Phenomics Facility, at the University of Adelaide, SA, Australia in three independent experiments performed from February to April 2016. A subset of 54 accessions were replicated twice in each experiment. The 54 accessions were selected based on seed availability and uniform germination. Each experiment consisted of 432 pairs of pots (378 and the 54 replicated accessions). Accessions were randomly assigned to each pair of pots, and water regimes were randomly assigned within pairs. Pairs were randomly partitioned in two smarthouses, each of which consisted of 24 lanes.

Seven days after transplantation (DAT) to soil, plants were thinned to one seedling per pot, and two layers of blue mesh were placed on top of the soil to reduce soil water evaporation. At 11 DAT, the plants were loaded on the imaging system and were watered to 90% FC. Water was withheld from one of the two pots for each accession beginning at 13 DAT. Water was withheld until the end of the experiment or until the FC reached 20%, after which the plants were maintained at 20% FC.

### Image analysis

The plants were imaged each day from 13 to 33 DAT using a visible [red–green–blue (RGB) camera; Basler Pilot piA2400-12 gc, Ahrensburg, Germany] from two side view angles separated by 90° and a single top view. The LemnaGrid software was used to extract ‘plant pixels’ from RGB images. The image analysis pipeline is identical to that described in Campbell *et al.* (2018). ‘Plant pixels’ from each of the RGB images for each plant and time point were summed and were used as a proxy for shoot biomass, which is referred to as projected shoot area (PSA). Several studies have shown that this metric is an accurate representation of shoot biomass ([Bibr CIT0016][Bibr CIT0008]; Knecht *et al.*, 2016). Outlier plants at each time point were detected for each trait using the 1.5× interquartile range rule. Potential outliers were plotted and inspected visually, and those that exhibited abnormal growth patterns were removed prior to downstream analyses. In total, 34 plants were removed, leaving 2558 plants for downstream analyses. Since the genome-enabled crop growth model does not accommodate missing data, accessions with missing values were excluded from further analyses, resulting in a total of 349 accessions being used for downstream analyses.

### Modeling shoot growth as a function of time and soil water content

The Gompertz growth model has been used extensively to model asymptotic processes that exhibit a sigmoid trend (Winsor, 1932). The classical Gompertz model is given by $PSA(t)=PSA_{max}e^{-e^{-r(t-T_{o})}}$, where *t* is a vector of time values, *r* is the absolute growth rate, PSA_max_ is the maximum biomass (e.g. asymptote), and *T*_0_ is the inflection point in the growth curve where the relative growth rate begins to slow. For the drought conditions imposed in the current study, we expect shoot growth to follow an exponential trajectory during the initial time points when soil water is not limiting. However, as the soil dries out, the growth rate should slow and, eventually, when soil water content falls below some threshold, growth should cease completely. Thus, the basic framework provided by the Gomertz model should capture these expected patterns.

To model the effects of water deficit on shoot growth trajectories, we devised a growth model that is an extension of the classical Gompertz growth model. The Gompertz growth model was modified so that shoot growth trajectories were modeled as a function of time and soil water content. This model is referred to as the WSI-Gomp model in the remainder of this manuscript. The WSI-Gomp model is given by

$$\begin{array}{l} \begin{array}{l} PSA(t)=PSA_{max}e^{-e^{-r(t-WSI^{\alpha })}} \end{array} \end{array}$$

where PSA_max_ is a parameter that describes the maximum biomass achieved by the plant; *r* describes the absolute growth rate; *t* is a vector of standardized time values [0,1]; and α is a genotype-specific tuning parameter that modifies the effect of WSI on PSA. WSI is the water stress index, a unitless index that describes the severity of water stress, and is given by

$$\begin{array}{l} \begin{array}{l} WSI=\frac{FC_{t}-FC_{Crit}}{FC_{Opt}-FC_{Crit}} \end{array} \end{array}$$

**Fig. 1.**
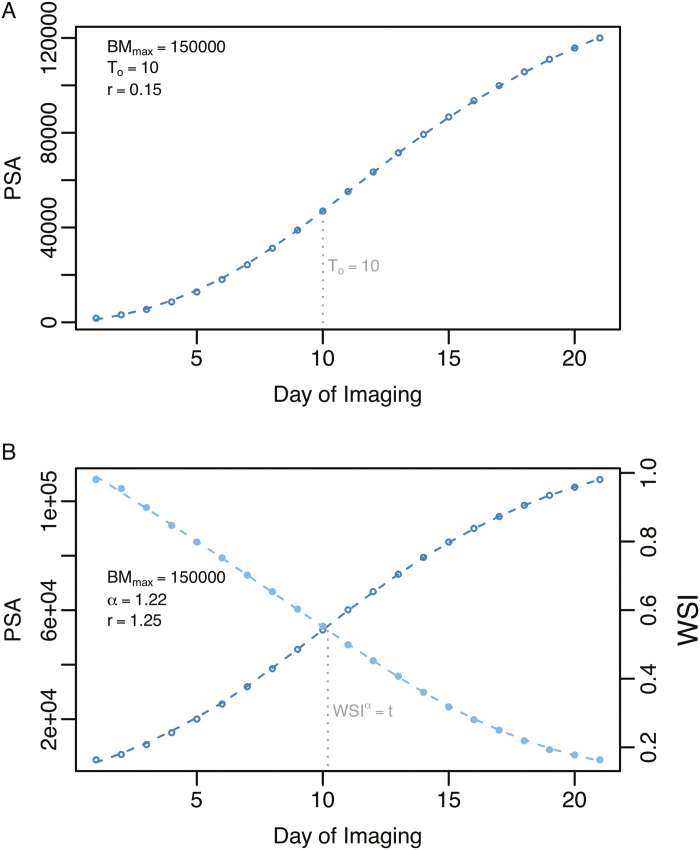
Graphical representation of the classical Gompertz model and the WSI-Gomp model. (A) The classical Gompertz growth model was used to generate PSA values over a 21 d period. The parameter values used are provided in the top left corner of the plot. The gray, vertical broken line indicates the inflection point (*T*_0_). (B) The WSI-Gomp growth model was used to generate PSA values over a 21 d period. PSA values are shown using dark blue points and a broken line. The light blue points and line indicate the WSI values over the 21 d period. WSI was calculated from simulated soil water content values that are typically of those experienced by water-stressed plants in the current study. The gray, vertical broken line indicates the inflection point (*T*_0_).

FC_*t*_ is the portion of FC at time *t*; FC_Crit_ (critical FC) is the proportion of FC in which growth ceases; and FC_Opt_ is the proportion of FC that is optimal for growth. FC was calculated at each time point from pot weights given by the automated watering system. Since FC_Crit_ and FC_Opt_ are unknown and likely to be genotype dependent, we assumed that the optimal conditions for growth in rice occur when the soil is completely saturated (i.e. FC_Opt_=1), and FC_Crit_ is equal to 0.1. Although these assumptions require empirical evidence to validate, they provide a standardized metric that describes soil water content in a decreasing non-linear trend that is on the same scale as the standardized time values. These characteristics allow PSA to be modeled as a function of time and soil water content using the Gompertz growth model. Figure 1 provides a graphical summary of the classical Gompertz and WSI-Gomp growth models for simulated soil water content values that would be typical for water-stressed plants in the current study, and Supplementary Fig. S1 at *JXB* online shows the effects of varying model parameters on shoot growth trajectories.

### Leveraging whole-genome regression to estimate model parameters

The ‘integrated approach’ developed by Onogi *et al.* (2016) uses a hierarchical Bayesian framework to simultaneously infer growth model parameters and marker effects. The models were fit using the R package GenomeBasedModel (Onogi, 2020; https://github.com/Onogi/GenomeBasedModel). Briefly, solutions for the growth model parameters are regressed on genome-wide markers, and extended Bayesian LASSO (least absolute shrinkage and selection operator; EBL) is used to predict marker effects for each of the model parameters. The regression model is given by

$$\begin{array}{l} y=\mu +W\beta \;+e \end{array}$$

**W** is an *n*×*m* matrix of marker genotypes coded as –1, 0, 1, *n* is the number of accessions (349), and *m* is the number of markers (33 697); μ is the intercept for each parameter; and β is an *m*×1 vector of predicted marker effect for each model parameter. Markers were obtained from RiceDiversity.org and have been described by Zhao *et al.* (2011). The prior distribution of marker effects for marker *i* is

$$\begin{array}{l} \begin{array}{ll} \beta_{i} & ∼N(0,\frac{1}{\tau_{0}^{2}\tau_{i}^{2}})\\ \tau_{i}^{2} & ∼inverse-gamma(1,\frac{\delta^{2}\eta_{i}^{2}}{2})\\ \delta^{2} & ∼gamma(\phi ,\omega )\\ \eta_{i}^{2} & ∼gamma(\psi ,\theta ) \end{array} \end{array}$$

τ _*i*_^2^ is the precision for the effect of marker *i*; η _*i*_^2^ is the marker-specific shrinkage parameter for marker *i*; δ ^2^ is the global shrinkage parameter; and ω, ϕ, θ, and ψ are hyperparameters. Default values were used for hyperparameters. We assume the following for WSI-Gomp model parameters

$$\begin{array}{l} \begin{array}{ll} PSA_{max} & ∼N(\mu_{PSA_{max}}+W\beta_{PSA_{max}},\frac{1}{\tau_{0,PSA_{max}}^{2}})\\ r & ∼N(\mu_{r}+W\beta_{r},\frac{1}{\tau_{0,r}^{2}})\\ \alpha & ∼N(\mu_{\alpha }+W\beta_{\alpha },\frac{1}{\tau_{0,\alpha }^{2}}) \end{array} \end{array}$$

τ ^2^_0,*p*_ is the residual precisions for model parameter *p*. Moreover, for the residuals, we assume N (0, 1/ τ ^2^_0_)]. With the ‘integrated approach’ proposed by Onogi *et al.* (2016), model parameters are inferred using a variational Bayes approach in which means and variances of the growth model parameters are obtained using Markov chain Monte Carlo sampling and are used to update EBL parameters.

### Genome-wide association for time of inflection

We sought to utilize the WSI-Gomp model to identify genomic loci that influenced the timing of the transition to a declining growth rate. To this end, we used model parameters obtained from the hierarchical Bayesian approach described above and observed WSI values to solve for the time of inflection (TOI). In the classical Gompertz growth model, $PSA(t)=PSA_{max}e^{-e^{-r(t-T_{o})}}$, the growth rate begins to decline when the (*t*–*T*_0_) term becomes positive (i.e. when *t* exceeds *T*_0_). Thus, *T*_0_ can be defined as the time of inflection. In the WSI-Gomp model, this component is given by (*t*–WSI ^α^). Thus, the TOI occurs when *t≥*WSI ^α^. Using the hierarchical Bayesian approach, we obtained estimates for α for each accession in drought and control conditions, and used these values to solve for TOI using WSI values for each accession in each experiment. TOI was defined as the first day in which (*t*–WSI ^α^) was positive. This yielded a single TOI value for each accession in each experiment.

These TOI values were used as a derived phenotype for further genome-wide association study (GWAS) analysis. The following Bayesian LASSO regression model was fit using the BGLR package (Pérez and Los Campos, 2014)

$$\begin{array}{l} y=X\beta \;+Za+e \end{array}$$

where **y** = **X** is an incidence matrix relating the vector **β** of fixed effects for experiment to observations, **Z** is an incidence matrix relating the vector of random marker effects **a** to **y**, and **e** is the residual. Since the vector **y** is a vector of discrete TOI values, **y** was treated as an ordinal response, and a probit link function was used. In Bayesian LASSO, the marginal prior distribution for each marker effect is a double exponential function that includes an unknown parameter λ ^2^ with a prior distribution λ ^2^~gamma (*r*, *s*) (Pérez and Los Campos, 2014). BGLR sets *s*=1.1 by default and solves for *r* based on the prior *R*^2^ of the model. Details on the BL approach implemented in BGLR is provided in the package vignette. A Gaussian prior with mean zero and variance equal to 1×10^10^ was used for fixed effects.

## Results

### Image-based phenotyping captures the sensitivity of rice to drought stress

**Fig. 2.**
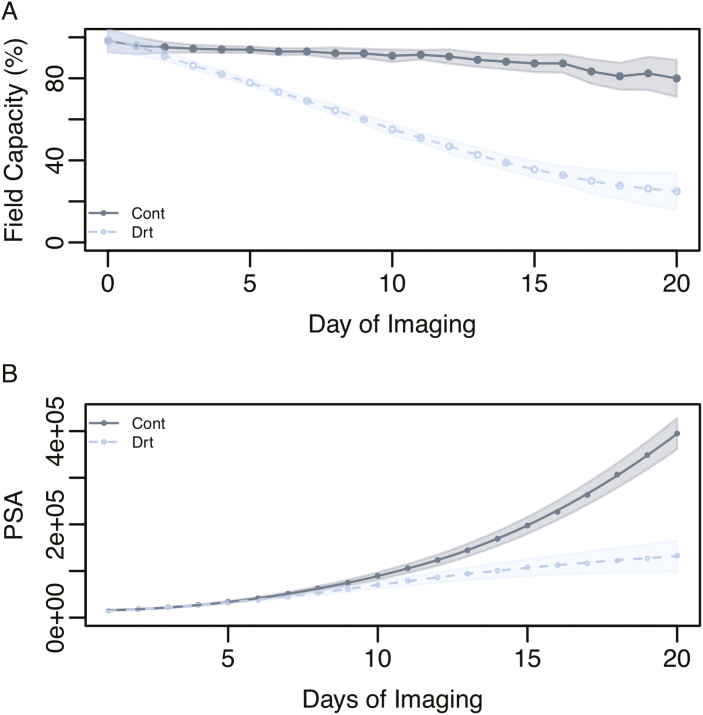
Effect of water deficit on shoot growth. (A) Mean percentage field capacity in drought and control conditions over the 21 d of imaging. (B) Mean shoot growth trajectories (PSA) in drought and control conditions over 21 d of imaging. Water was withheld starting at day 1 of imaging. The shaded regions indicate the SD for each treatment.

To examine drought responses in rice (*Oryza sativa*), a diversity panel was phenotyped over a period of 21 d during the early vegetative stage using an automated high-throughput phenotyping platform (Supplementary Dataset S1). Control plants were maintained at 90% FC, while water was withheld from drought-treated plants until a final FC of 20% was reached. A *t*-test was carried out at each time point to determine when a significant reduction in soil water availability was experienced. A significant difference in pot water content (FC) was observed from the second day of imaging (Fig. 2A; *P*<0.0024, Bonferroni’s correction with α =0.05) when the drought plants on average were at 90.9% FC.

The impact of drought stress on shoot growth (biomass) was estimated from RGB images and expressed as a digital metric called PSA. An ANOVA was carried out at each time point using the a linear model that included main effects for treatment and accession and the interaction between treatment and accession. Significant effects for the interaction between accession and treatment were observed from day 16 onward. Drought treatment had a significant effect on PSA beginning on the fourth day of imaging (Fig. 2B; *P*<0.0024, Bonferroni’s correction with α =0.05). Interestingly, at this time point drought-treated plants, on average, were at 81.9% FC, which is only ~12.5% below control plants. These data suggest that even a small limitation of water can have a significant impact on shoot growth in rice, thus confirming the high level of drought sensitivity reported for rice (Lafitte *et al.*, 2004).

### Defining the growth model

The Gompertz growth model has been used extensively to model asymptotic processes that exhibit a sigmoid trend (Winsor, 1932). This sigmoid/asymptotic trend is to some degree visible in the mean growth trajectory in Fig. 2B. While the classical Gompertz model provides an intuitive framework to model asymptotic growth trajectories as a function of time, it does not accommodate environmental data, and therefore cannot be used to address how shoot growth varies in response to soil water content.

To address this limitation, we sought to modify the Gompertz growth model so that shoot growth trajectories could be modeled as a function of time and soil water content. We defined an index (water stress index, WSI) from daily records of soil water content for each plant that reflects the severity of water stress. WSI is given by $WSI=\frac{FC_{t}-FC_{Crit}}{FC_{Opt}-FC_{Crit}}$, where FC_*t*_ indicates the percentage FC at time *t*, FC_Opt_ is the optimal percentage FC for growth, and FC_Crit_ is the percentage FC at which growth ceases. Since these values are expected to vary depending on the genotype, we assumed that growth will cease at 10% FC (FC_Crit_=1) and the growth will proceed optimally when the soil is saturated (FC_Opt_=100). This equation provides a unitless metric that will vary between 0 and 1, with higher values indicating lower water stress and lower values indicating a greater stress. For this metric to be introduced into the Gompertz growth model, we standardized the time values so that they ranged from 0 to 1. Finally, we introduced a third parameter (α) into the model that acts as a genotype-dependent tuning parameter and modifies the effect of WSI on growth rate. This new WSI-integrated model (WSI-Gomp) is given by $PSA(t)=PSA_{max}e^{-e^{-r(t-WSI^{\alpha })}}$. The WSI-Gomp model is shown in Fig. 2B.

**Fig. 3.**
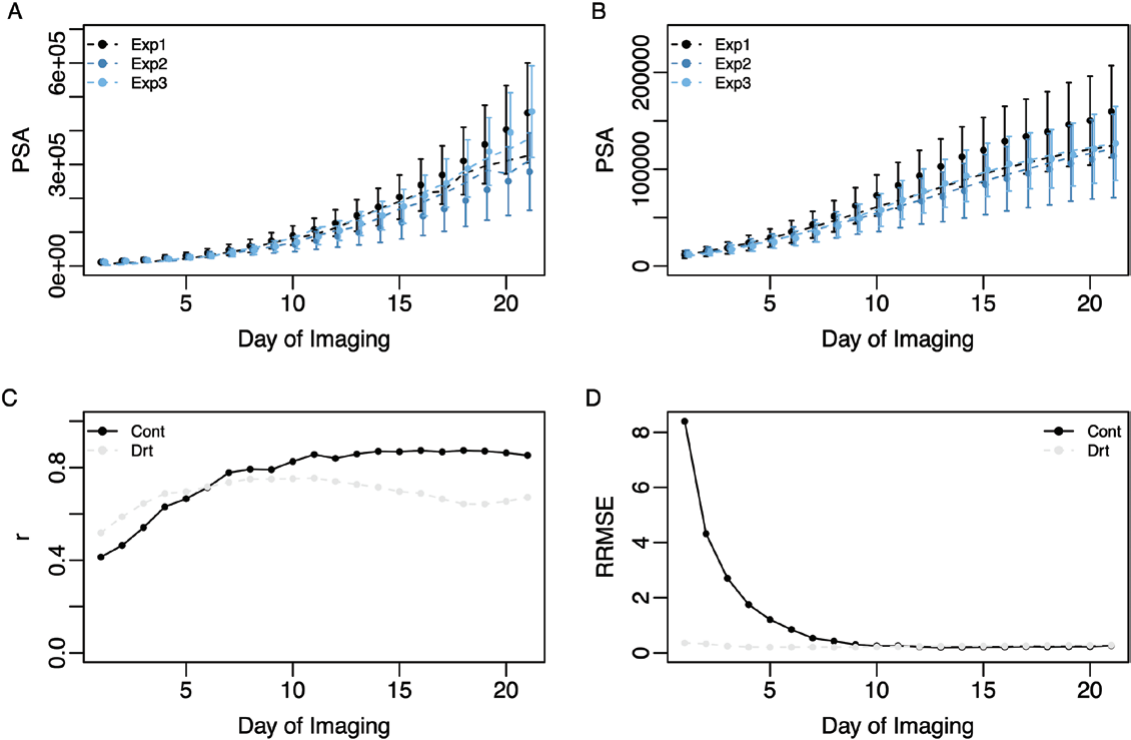
Capturing shoot growth trajectories using the WSI-Gomp model. Observed (points) and predicted (broken line) mean shoot growth trajectories for each experiment under control (A) and drought (B) conditions. For both (A) and (B), Nelder–Mead optimization was used to fit the WSI-Gomp model to the mean shoot growth trajectories for each experiment. (C) Average correlation between predicted trajectories and observed PSA values. (D) Relative root mean squared error (RRMSE) between predicted trajectories and observed PSA values. RRMSE was calculated as RMSE at time *t* divided by the mean predicted values at time *t*. For both (C) and (D), the WSI-Gomp model was fit using the hierarchical Bayesian model, and predicted PSA values were compared at each time point with observed values for each accession.

To capture the effects of soil water deficit on growth trajectories, the WSI-Gomp model was fit to growth trajectories in drought and control conditions for each of the 349 accessions using a hierarchical Bayesian approach that leverages the genetic relationships among lines to obtain solutions for the model parameters (Onogi *et al.*, 2016). Model parameter estimates for each accession were used to predict growth trajectories employing observed WSI values. The ability of the WSI-Gomp model to capture these dynamic responses was assessed by comparing predicted PSA values and observed values at each time point using two metrics: root mean squared error (RMSE) and Pearson’s correlation. Overall, the WSI-Gomp model provided a good fit to the observed shoot growth trajectories (Fig. 3). The correlation between observed and predicted PSA values ranged from 0.41 to 0.87 in the control, while the correlation was slightly lower in drought conditions and ranged from 0.52 to 0.75. Correlation values were lowest for early time points in both control and drought conditions, suggesting that predictions for these time points may be inaccurate. However, at later time points, there was a high agreement between predicted and observed values for PSA. Collectively, these results suggest that the WSI-Gomp captures shoot growth trajectories in contrasting water regimes; however, other factors not accounted for in the growth model also influence observed PSA values.

### Leveraging the growth model for biological inference

**Fig. 4.**
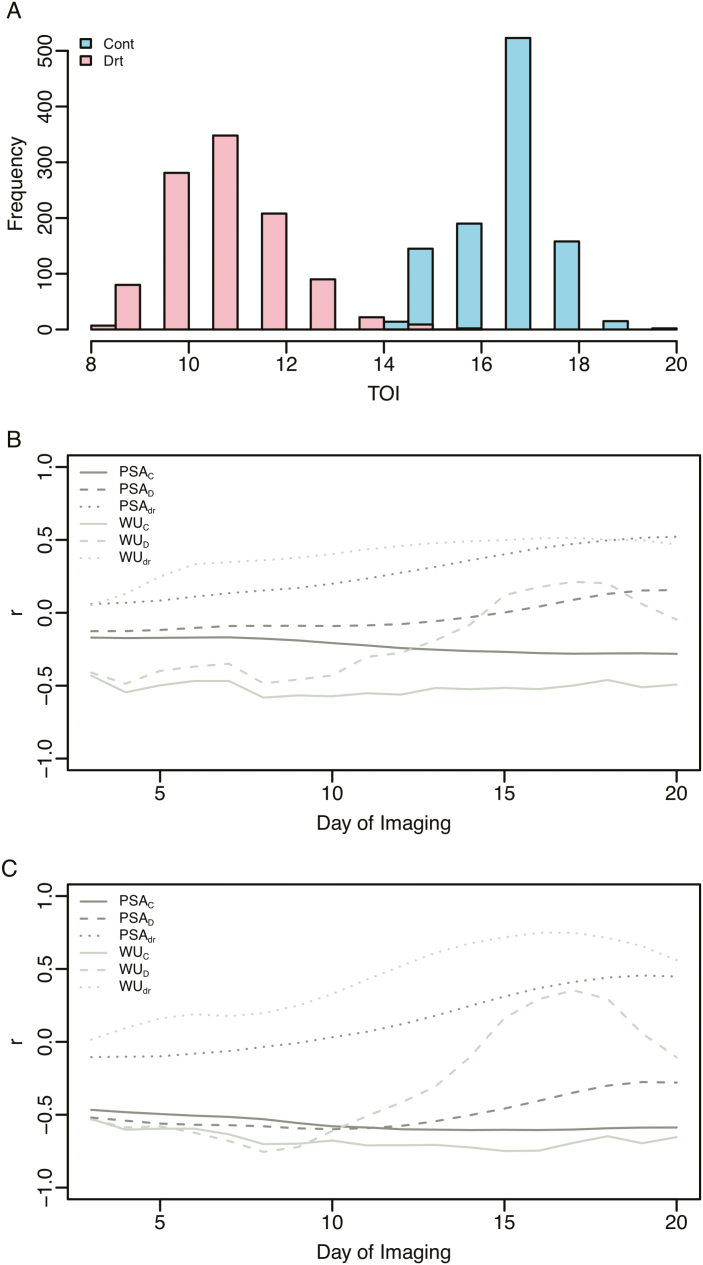
Distribution and interpretation of predicted time of infection. (A) The distribution of time of inflection (TOI) values in control and drought conditions. Correlation between time of inflection in control (B) and drought (C), and empirical observations for projected shoot area (PSA) and water use (WU). WU for a given day is calculated as the difference in soil water content from the previous day and soil water content on the current day. In cases where the plant received water (e.g. control plants), pot weights after watering were used to calculate soil water content values for the previous day, and pot weights prior to watering were used for soil water content values for the current day. Spearman’s correlation was performed using a 3 d sliding window.

The WSI-Gomp model provides a means to model PSA trajectories as a function of declining soil water content and allows the inflection point in growth curves to be estimated using observed WSI values. With this in mind, we next sought to determine what observable characteristics influence the timing of this inflection point in drought conditions. To this end, we calculated the TOI for each plant in drought by determining the earliest time in which the (*t*–WSI ^α^) component of the model became positive (Supplementary Dataset S2). As expected, the predicted TOIs were lower in drought conditions compared with control, indicating that the inflection of the growth curve occurs early under drought conditions compared with well-watered conditions (Fig. 4A). TOI in drought-treated plants ranged from 8 d to 16 d of imaging, while in control plants TOI values ranged from 14 d to 20 d.

To determine how observable phenotypes influenced TOI, the predicted TOI values were compared with WU, PSA, and the ratio of these values in drought and control (indicated by the subscript ‘dr’ meaning drought response) over the course of the experiment. Relationships were assessed using Spearman’s correlation with a 3 d sliding window (Fig. 4B, C). In drought conditions, we observed a negative relationship between TOI in drought (TOI_D_) and PSA in both control and drought conditions (PSA_C_ and PSA_D_, respectively), indicating that larger plants tend to have earlier retardation of shoot growth rate (Fig. 4B). The relationship between TOI_D_ and PSA_D_ became weaker as the soil water declined and drought became more severe. This trend is probably because at these time points shoot growth in large plants was likely to have already been repressed by drought. Similar, albeit slightly stronger, negative correlations were observed between WU in control (WU_C_) and TOI in drought (TOI_D_). An interesting trend was observed for WU_D_ and TOI_D_. At early time points (e.g. days 0–14) a negative correlation was observed between TOI_D_ and WU_D_. However, around days 15–18, this trend is reversed completely, with a positive correlation observed between WU_D_ and TOI_D_. As expected, TOI_D_ showed a positive relationship with PSA_dr_ (i.e. the ratio of PSA in drought to control), indicating that accessions with early inflection points tend to show a larger reduction in PSA under drought relative to control.

Similar trends were observed in control conditions; however, the values of the correlation coefficients were different compared with drought (Fig. 4C). A negative relationship was observed between TOI in control (TOI_C_) and PSA in control (PSA_C_), which is consistent with the relationship observed for TOI_D_ and PSA. However, TOI_C_ and PSA_C_ showed a very weak relationship, with a slight negative correlation during initial time points and a very weak positive relationship observed at later time points. Consistent with drought conditions, the relationship between WU_C_ and TOI_C_ showed a strong negative correlation. Moreover, the correlation between TOI_C_ and WU_D_ was negative at early time points and positive at later time points, which is similar to the trend observed between TOI_D_ and WU_D_. Although the interpretation of α and TOI in control conditions is not very straightforward because plants were grown in the absence of water stress, the observed correlation suggests that these parameters may have a similar interpretation to that in drought conditions.

### Genome-wide association provides insight into loci influencing shoot growth trajectories

**Fig. 5.**
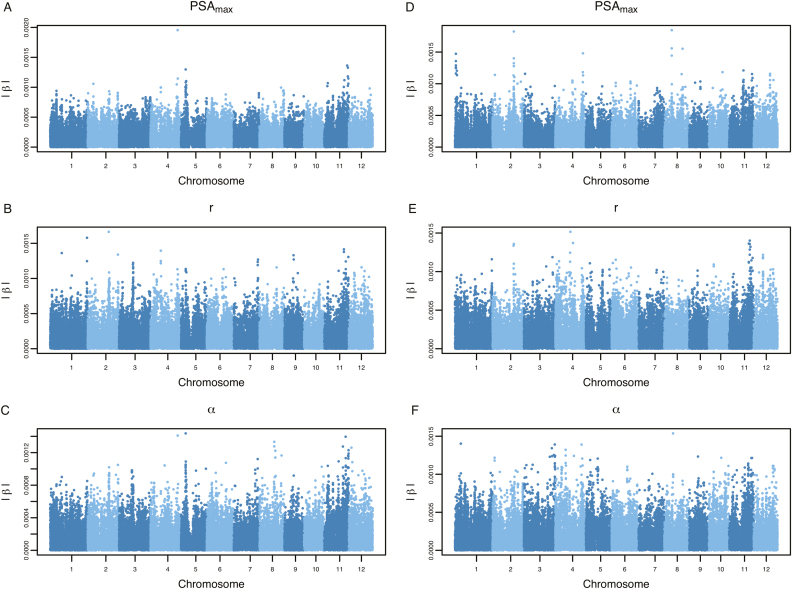
Genomic regions influencing model parameters. Predicted marker effects are shown for each of the WSI-Gomp model parameters. (A–C) Marker effects for model parameters fit to growth trajectories in control conditions; (D–F) marker effects for drought conditions. The absolute value of predicted marker effects (|β|) is shown on the *y*-axis.

Model parameter estimates for the WSI-Gomp model were obtained using a hierarchical Bayesian framework, wherein the growth model is fit in the first level, and in the second level an EBL approach is used to predict marker effects from model parameters. Thus, this information can be leveraged to identify QTLs and potential candidate genes that may influence shoot growth trajectories in response to water deficit. To this end, we sought to utilize the inferred marker effects to identify genomic regions that regulate model parameters and influence dynamic shoot growth trajectories in response to water availability. The absolute values of inferred marker effects are provided in the Manhattan plots in Fig. 5 and Supplementary Dataset S3. Since obtaining *P*-values from Bayesian approaches is non-trivial, we report loci and candidate genes for the top-20 single nucleotide polymorphisms (SNPs) ranked based on the absolute value of marker effects (|β|).

The model parameters *r* and α in both control and drought conditions exhibit a polygenic genetic architecture. We identified several markers with small contributions to the parameter values. Although the model parameters α and *r* showed a polygenic architecture, several notable genes were identified within the regions defined by SNPs with relatively larger effects (Supplementary Dataset S4). For instance, at ~6.7 Mb on chromosome 1, a gene encoding an osmotin protein (*OSM34*) was found ~75 kb upstream of the top SNP associated with α in drought within this region. Osmotin proteins play a role in plant biotic and abiotic stress responses, particularly drought stress (Narasimhan *et al.*, 2009; Sharma *et al.*, 2013). Additionally, a membrane-bound protein involved in chilling tolerance, *COLD1*, was found ~27 kb downstream of the SNP with the largest effect on chromosome 4 for α in drought (Ma *et al.*, 2015). The presence of these two genes known to be involved in abiotic stress responses warrants further investigation.

The parameter PSA_max_ showed a simpler genetic architecture in control and drought conditions. In control conditions, one large QTL was identified on chromosome 4 with the SNP, with the largest effect located at ~31.4 Mb on chromosome 4. Within this region, a gene involved with the regulation of polar auxin transport, *Narrow Leaf1* (*NAL1*), was identified. Several studies have reported that variants in the *NAL* gene have pleiotropic effects and alter plant vascular patterning, spikelet number, leaf size, root system architecture, and shoot biomass (Qi *et al.*, 2008; Fujita *et al.*, 2013). In drought conditions, several QTLs were identified for PSA_max_, with notable peaks located on chromosomes 1, 2, 4, and 8. The SNP with the largest effect was located at ~21 Mb on chromosome 8. Within this region, a gene known to regulate flowering time under short-day conditions was identified, *GF14c* (Purwestri *et al.*, 2009). Moreover, a second gene known to influence biomass and seed size, *OsMPS*, was identified on chromosome 2 at ~24.5 Mb (Schmidt *et al.*, 2013). Since PSA_max_ is a parameter that describes the maximum biomass for each accession, the presence of genes known to regulate flowering time and biomass is promising and suggests that this parameter is biologically meaningful.

### Elucidating the genetic loci influencing time of inflection in contrasting water regimes

**Fig. 6.**
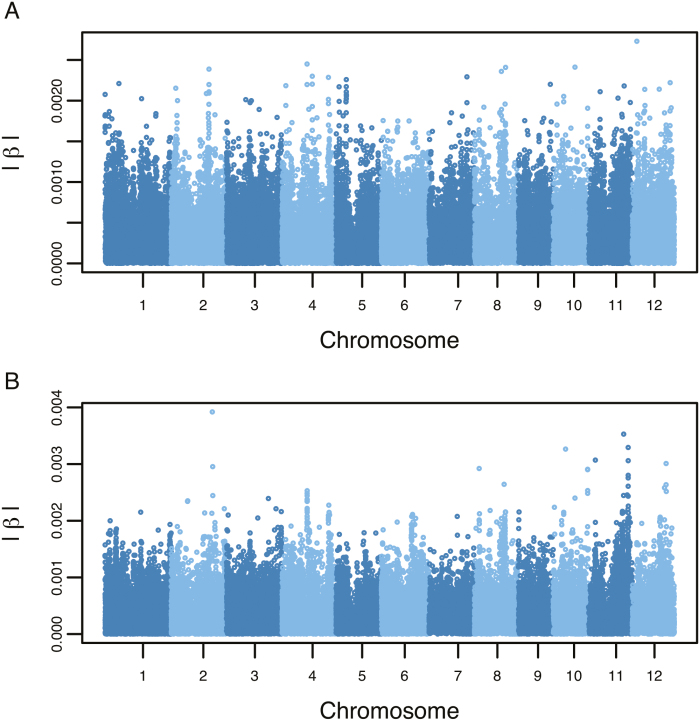
Manhattan plots for time of inflection (TOI). GWAS was conducted using TOI values in control (A) and drought conditions (B). Each point indicates an SNP marker, and the *y*-axis shows the absolute value of predicted marker effects (|β|).

In addition to the parameters explicitly defined by the model, TOI can also be considered an additional phenotype that can be analyzed using conventional GWAS frameworks. With this in mind, we sought to identify QTLs that were associated with TOI using a Bayesian whole-genome regression approach (Supplementary Dataset S3). Estimates for model parameters were combined with observed environmental covariates to solve for the TOI for each accession in drought and control conditions. Marker associations with TOI were assessed using a GWAS approach that accounted for the ordinal response variable, and results are discussed in the context of the top-20 ranked SNPs based on |β| (Fig. 6).

GWAS for TOI in control conditions showed that many SNPs have a small effect on the phenotype, indicating a complex genetic architecture for TOI in control conditions (Fig. 6A). However, for drought conditions, GWAS revealed two notable regions characterized by SNPs with relatively larger effects (Fig. 6B). The first peak was identified at ~27 Mb on chromosome 2, while the second peak was located at 22.9 Mb on chromosome 11.

## Discussion

Drought tolerance during the vegetative growth stage is most simply defined as the ability to maintain growth under water deficit. It is determined by the amount of water available to the plant and how efficiently the water is used to gain biomass. In terminal drought environments, where a fixed amount of water is available during the early season, the ability to maintain growth will be dependent on how well the plant can manage these resources throughout the season. Thus, when studying drought tolerance, especially in terminal drought environments, it is important to jointly consider these factors. In the current study, we imposed a severe drought stress by completely withholding water for a period of 20 d (or until pots reached 20% FC). The effects of this severe stress were apparent soon after withholding water, as drought-stressed plants showed a significant reduction in shoot biomass after 4 d compared with control plants (Fig. 2).

Given the importance of accounting for water availability when modeling temporal shoot growth trajectories, we developed a growth model that jointly models shoot biomass and soil water content. While the model parameters themselves can be used to describe characteristics of the growth curve and provide insight into the processes that influence shoot growth, the model can also be leveraged for additional biological inferences. For instance, we used genotype-specific parameter estimates to determine the point in which the growth rate begins to decline (i.e. TOI). While this information can also be obtained with the classical Gompertz growth model, the WSI-Gomp model leverages both time and temporal soil water availability while the former only utilizes time. Since the time values are standardized to be on the same scale as the WSI with the WSI-Gomp model, this metric can be interpreted in two ways: (i) the time in which the growth rate begins to decline; or (ii) the soil water content value that begins to repress growth. Regardless of the interpretation, this approach provides a means to assess drought sensitivity while accounting for variation in soil water content between plants.

### Joint modeling suggests a trade-off between vigor and drought tolerance

The TOI provided biological insight into the relationship between plant size or vigor and morphological responses to a severe water deficit. Temporal correlation analyses between TOI and morphological and physiological responses revealed that large, vigorous plants tend to have an earlier decline in growth rate under severe drought conditions (Fig. 4). Moreover, these plants tend to have high water demands in control conditions, and quickly exhaust soil water resources in drought conditions. The link between early vigor and drought responses has been studied extensively. Although some studies suggest that early vigor is advantageous in drought-prone environments, these benefits are highly dependent on the type of drought stress that is prevalent in these regions (Tardieu, 2011). A study by Kamoshita *et al.* (2004) evaluated six rice accessions under short and prolonged drought and examined the relationship between root system architecture, osmotic adjustment, and biomass production. They found that highly vigorous accessions rapidly developed a dense root system and extracted water quickly, but were also more sensitive to prolonged drought stress compared with low vigor genotypes. However, these plants tended to recover more quickly after rewatering compared with low vigor accessions. A more recent study by Rebolledo *et al.* (2012) found similar results and suggested that vigorous accessions also quickly exhaust starch reserves under prolonged drought, resulting in a greater decline in biomass production compared with less vigorous accessions. Collectively, these studies support the observed negative correlation between plant size and drought sensitivity (as assessed with TOI), and suggest a trade-off between vigorous growth and the maintenance of growth in prolonged drought stress. Further studies are necessary to determine whether these relationships can be decoupled, or to identify the optimal balance between these two attributes.

### Leveraging the genome-enabled growth model for candidate gene discovery

The hierarchical Bayesian framework developed by Onogi *et al.* (2016) provides a powerful approach to improve the estimation of model parameters and to estimate the genomic contributions to the model parameters. Since the model parameters are regressed on genome-wide SNP markers, this framework provides a means to identify important loci that influence trait trajectories (i.e. GWAS). While the initial study by Onogi *et al.* (2016) showed both applications of the approach, their primary objective was genomic prediction. Here, we leveraged the genome-enabled growth modeling approach to identify genomic regions that influence dynamic drought responses.

Many of the model parameters show a complex genetic architecture characterized by many loci with small effects (Fig. 5). However, several notable regions exhibiting relatively large effects were identified that harbored potential candidate genes. For instance, two notable peaks were identified on chromosomes 1 and 4 for the parameter α in drought conditions (Fig. 5F). Both regions harbored candidate genes that have been reported to regulate drought and/or osmotic stress responses in plants. The region on chromosome 4 harbored a gene that is known to regulate chilling tolerance in rice, *COLD1* (Ma *et al.*, 2015). *COLD1* was shown to be involved with the Ca^2+^ signaling response to cold stress. In Arabidopsis, the *COLD1* orthologs, *GTG1* and *GTG2*, are membrane-bound abscisic acid receptors (Pandey *et al.*, 2006, 2009). However, *COLD1* exhibits GTPase activity that is absent in *GTG1/2* (Ma *et al.*, 2015). Thus, further studies are necessary to determine whether *COLD1* participates in drought responses.

In addition to the candidate genes associated by model parameters, whole-genome regression performed with TOI in drought conditions revealed a potential role for additional genes in the genetic regulation of the timing of growth responses to drought (Fig. 6). An aquaporin gene, *OsPIP1;1*, was identified within a prominent peak on chromosome 2 associated with TOI in drought conditions. Aquaporins are a large family of proteins that were initially reported to act as water transporters, but have since been shown to also transport CO_2_ and H_2_O_2_ (Dynowski *et al.*, 2008; Uehlein *et al.*, 2003; Bienert and Chaumont, 2014; Maurel *et al.*, 2015; Wang *et al.*, 2016; Rodrigues *et al.*, 2017). Aquaporins have received considerable attention as a potential target to modify whole-plant water transport and improve water status during drought stress (Sadok and Sinclair, 2009; Devi *et al.*, 2012; Choudhary and Sinclair, 2014; Schoppach *et al.*, 2014; Grondin *et al.*, 2016). Work by Grondin *et al.* (2016) showed that aquaporins account for ~85% of root hydraulic conductivity in rice under drought stress, and demonstrated that the expression of *PIP1;1* is induced by drought stress.

### Concluding remarks

Improving drought tolerance in rice is a challenging objective. Efforts to improve drought tolerance are hindered by the heterogenity in drought-prone environments, the breadth and complexity of traits underlying drought adaptation, and the difficulty in characterizing large populations for these traits. Recent advances in phenotyping technologies have provided an effective means to measure morpho-physiological traits frequently throughout the growing season, and provide plant breeders and geneticists with dense phenotypic data describing complex responses. However, these technological advances must be coupled with frameworks that accommodate these multidimensional data sets, while providing a means to leverage high density genotypic data to predict phenotypes and novel biological inference. In this context, the genome-enabled growth model proposed is a significant advancement towards addressing this need. The WSI-Gomp model provides a simple, biologically meaningful framework that can describe complex temporal responses using few parameters. Moreover, since genome-wide markers are used to estimate model parameters, the inferred marker effects can be used to identify genes that may contribute to these responses, estimate genetic values for model parameters for known individuals, as well as predict the phenotypes for new, uncharacterized individuals. Thus, these data can both be leveraged for genetic inference of complex drought responses, and make selections based on model parameters. This study is the first to leverage a genome-enabled growth model for genomic inference in rice, and provides novel insights into the basis of dynamic growth responses to drought stress.

## Supplementary data

Supplementary data are available at *JXB* online.

**Fig. S1.** Predicted shoot growth trajectories from the WSI-Gomp model with varying model parameters.

**Dataset S1.** Raw phenotypic data for all 349 accessions used to fit the WSI-Gomp model.

**Dataset S2.** Model parameter and time of inflection estimates for all 349 accessions obtained from the WSI-Gomp model.

**Dataset S3,** Marker effects for GWAS for model parameters and time of inflection.

**Dataset S4.** Candidate genes for model parameters and time of inflection.

eraa280_suppl_Supplementary_Figure_S1

eraa280_suppl_Supplementary_Data_S1

eraa280_suppl_Supplementary_Data_S2

eraa280_suppl_Supplementary_Data_S3

eraa280_suppl_Supplementary_Data_S4

eraa280_suppl_Supplementary_Legends

## Data availability

All data and codes used in this study can be accessed at https://github.com/malachycampbell/RiceCGM/tree/master.

## Abbreviations

DAT: days after transplantation
FC: field capacity
Gomp: Gompertz
GWAS: genome-wide association study
LASSO: least absolute shrinkage and selection operator
PSA: projected shoot area
QTL: quantitative trait locus
RGB: red–green–blue
SNP: single nucleotide polymorphism
TOI: time of inflection
WSI: water stress index
WU: water use
