# Supplementary material for: Leveraging genome-enabled growth models to study shoot growth responses to water deficit in rice
Source: J Exp Bot. 2020 Jun 12;71(18):5669–79. doi: 10.1093/jxb/eraa280 (PMC7501813; doi:10.1093/jxb/eraa280)
Supplement: eraa280_suppl_Supplementary_Figure_S1 [file eraa280_suppl_supplementary_figure_s1.pdf]

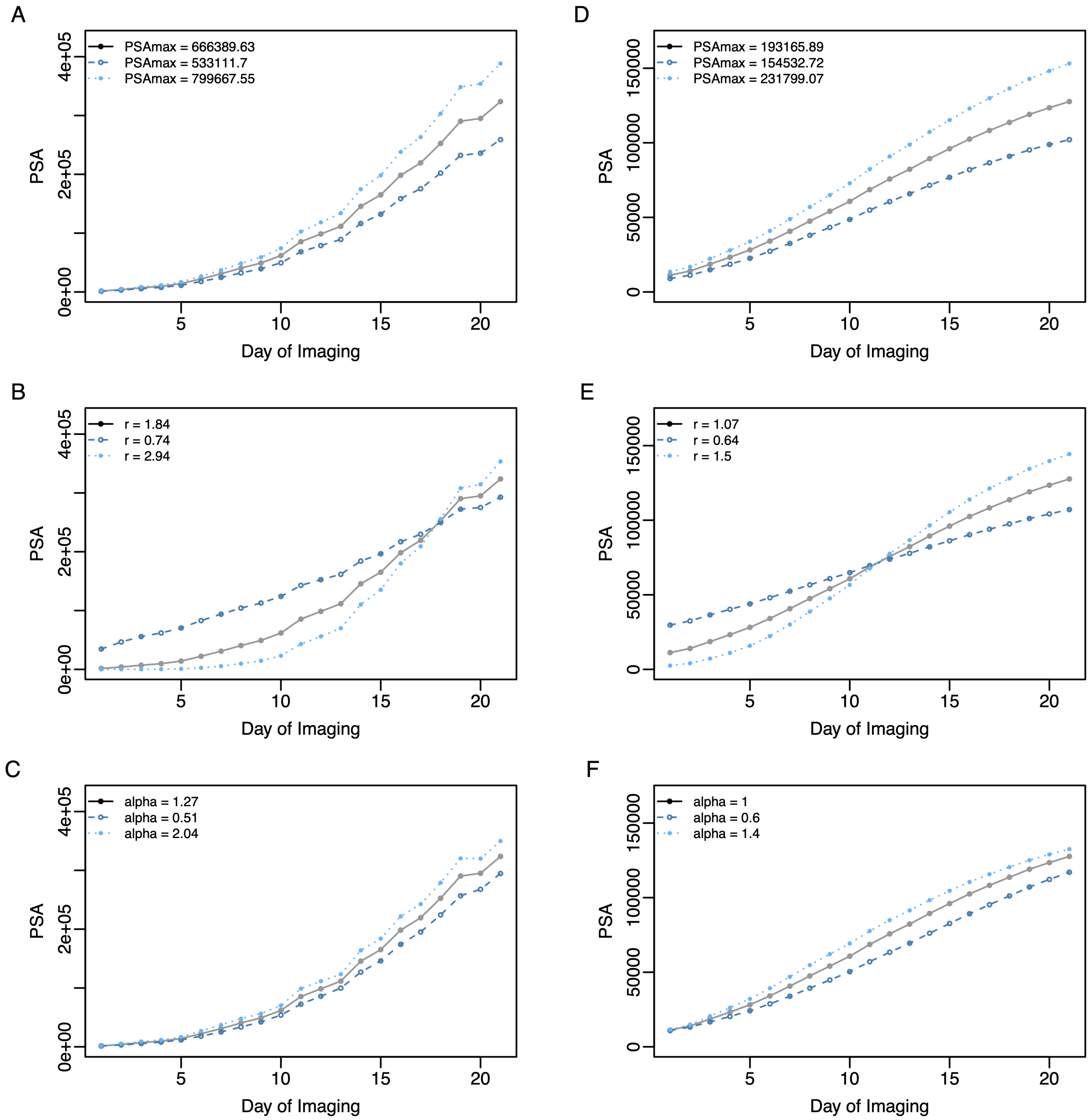

Figure S1. Predicted shoot growth trajectories from the WSI-Gompertz model with varying model parameters. Model parameters estimates and soil water content in control and drought conditions were obtained for a representative accession. Shoot growth trajectories were generated using the WSI-Gompertz model and soil water content measurements from the second experiment. Panels A to C show predicted shoot growth trajectories based on model parameter estimates and soil water content in control conditions, while panels D-F show predictions based on parameter estimates and environmental covariates in drought conditions.
