# Supplementary material for: Leveraging genome-enabled growth models to study shoot growth responses to water deficit in rice
Source: J Exp Bot. 2020 Jun 12;71(18):5669–79. doi: 10.1093/jxb/eraa280 (PMC7501813; doi:10.1093/jxb/eraa280)
Supplement: eraa280_suppl_Supplementary_Legends [file eraa280_suppl_supplementary_legends.docx]

# Supporting Information

- **Supplemental Figure S1:** Predicted shoot growth trajectories from the WSI-Gompertz model with varying model parameters.
- **Supplemental** Dataset **S1:** Raw phenotypic data for all 349 accessions used to fit the Gomp-WSI model.
- **Supplemental** Dataset **S2:** Model parameter and time of inflection estimates for all 349 accessions obtained from the Gomp-WSI model.
- **Supplemental** Dataset **S3:** Marker effects for GWAS for model parameters and time of inflection.
- **Supplemental** Dataset **S4:** Candidate genes for model parameters and time of inflection.
